# Harmony

**Published:** 1895-02

**Authors:** H. L. Ambler

**Affiliations:** Cleveland, O.


### THE DENTAL REGISTER.

Vol. XLIX.] FEBRUARY, 1895. [No. 2.

### COMMUNICATIONS.

### Harmony.

BY H. L. AMBLER, D.D.S., M D., CLEVELAND, O.

Read before the Ohio State Dental Society, December, 1894.

The poet says: "There is a bond which holds each cord in harmony." Can we find that bond? The practice of dentistry can never be separated from the human anatomy, thus the connection between dentistry and medicine must always be an intimate one, and both callings only exist in consequence of the ills to which the flesh is heir. "The true professional spirit is one of generosity," and if applied in co-operation and interchange of professional thought between M.D. and D.D.S., advancement for each will certainly result. Neither should try to be completely independent of the other, for all things being equal, the M.D. and D.D.S., who agree to rely upon and assist each other, will be successful in curing many cases, where, alone, either one would have failed. One result of consultation will be to alter their ideas and relations to each other, and also greatly aid in relieving suffering humanity. Co-operation, working together, and exchange of knowledge should be in private practice and before medical and dental societies. In the writings of Hippocrates, Galen, and Celsus, medicine, surgery and dentistry were all included, and history informs us that the ancient Egyptian surgeons, physicians and dentists worked in harmony. Their united efforts have furnished such an accumulation of knowledge that the work has been to a great extent classified and apportioned to each one, but this does not furnish sufficient and proper grounds for them to ignore each other. Harmony and mutal interest can be attained by closer association, for we are dependent upon the physician, and he is dependent upon us. Medicine or dentistry cannot be productive of as much good as when reasonably combined. If a dentist has no medical education, cases will occur in his practice which could sooner and easier be cured by consulting with an M.D. When a patient comes to you with a syphilitic, or any other lesion of the mouth which you fail to diagnose, it is much better for the patient and more to your credit to consult with an M.D. and have the proper treatment administered, than for you to prescribe a remedy or operation, the result of which you could only imagine. Here the M.D. might be able to give a correct prognosis, foreseeing what results would follow from a certain course of treatment. The M.D. should act in the same manner with his patients, for he comes to the dentist very quickly when he cannot cure his *own* toothache. As a general thing, when dental diseases—so-called—are discussed by physicians, or medical diseases—so-called—are discussed by dentists, it is shown that there is lack of knowledge by each party. There are M.D.'s who have given attention to some dental lesions, and there are dentists who have devoted study to some medical lesions, but they would be an exception to the foregoing statement. Only seven or eight medical colleges in the U. S. give any special attention to the teeth and their diseases, so that lack of technical knowledge makes the physician sometimes mistake effects for causes when the disease is of dental origin, thus suffering is prolonged and occasionally death results. The *Lancet* reports a case, viz: A patient was brought to a London hospital, in a weakened condition with an abscess on the neck, and after being treated a very short time died. At the post-mortem the surgeon testified that death resulted from gangrene of the lungs and exhaustion from the effects of extending alveolar abscess, followed by pyæmia, which was produced by an ulcerated tooth.

The M.D. who can contribute to the fund of dental knowledge, is the one who should be heartily welcomed at all times, and it is a duty we owe to ourselves and patients to learn all the good things we can from everybody, as our profession is one in which no scientific attainment is too great to be made use of. "By joining hands with the M.D. we can hope for better things, which will raise us from the age of selfishness, and place us upon an eminence which will enable us to derive light from all the bright luminaries which adorn the profession."

Some of the publications of the International Medical Congress and the American Medical Association are read with pleasure and profit by dentists, and some essays in dental journals we trust would be of assistance to the busy practitioner or surgeon. M.D.'s read essays before dental societies; we had the pleasure of listening to one at our last meeting, and dentists do the same before medical societies; still there is not enough of this kind of interchange, much more of it would bring about a better understanding. Neither individuals nor societies should place any barriers between the M.D. and D.D.S., for the time has come when neither can afford to disparage the other, and the sooner they come to this conclusion, the better for both. "Many times surgeons and dentists have to seek the assistance of each other in order to effect cures. Sometimes the dentist has to prepare the way for the surgeon's knife, and in other cases the dentist has to aid the surgeon by a mechanical appliance. It is no reflection upon the most skillful and distinguished surgeons to say that they ought in many cases to seek the aid of the dentist and his mechanisms to supplement their services in the interests of patients." The gratifying results which have been thus accomplished, have been well demonstrated in numerous cases. In the restoration of lost portions of either maxilla, whether from disease or surgical operation, the surgeon is quite ready to call in the skill of the dentist to construct some appliance which will remedy the deficiency, restore symmetry and usefulness. E. H. Angle, D.D.S., of Minneapolis, has been appointed surgeon to the Great Northern R. R. to treat all cases of fractured maxillæ. If, for any reason, staphylography is a failure, the dentist can give relief and comfort to the patient, by making them an obturator.

Infantile dentition is more or less a factor in fever, diarrhœa, enteritis, obstinate vomiting, spasmodic coughing and convulsions; in such cases we suggest that consultation might be of advantage, for sometimes tumefaction of the gums and local disturbances are not very well marked. The teeth and the mucous membrane of the mouth are so closely related to the pharynx, œsophagus, and intestines, that there is no reason why they should not cause disease. Those who have not made it a study cannot be expected to fully understand about the changes which are taking place during dentition, but from a scientific knowledge of the tissues with which the professional man comes in contact, he derives the art of producing them in health or disease. All will be ready enough to admit that the more one knows of any and all organs of the human subject, the easier it will be for them to meet the formidable array of complicated diseases which may be presented.

Those who are too erudite to ever find anything new in the unlimited field of medicine or dentistry should soon be called to a higher sphere where they *may* have room for boundless expansion. Sometimes we find otalgia produced by reflex irritation of the dental nerves.

The important vascular and nervous ties binding together teeth and eyes show clearly enough that a certain pathological relationship between those organs is no more than might be expected, while the knowledge we possess concerning reflex affections in other parts of the body must incline us to a belief in the reality of such a connection. Examination of the teeth should never be neglected in treatment of diseases of the eye, and the removal of any morbid dental condition should always be aimed at. Gazelowsky claims that the following diseases stand in casual connection with carious teeth: reflex asthenopia, closely related to caries of the anterior molars, inflammation of the cornea, iris, and of the inner membranes, through the transmission of a "kind of ascending neuritis" by the ciliary nerves, iridio choroiditis, reflex dilatation of the pupil, blepharospasmus and cramp of the ocular muscles, exophthalmus from transmission of inflammation through the maxillary sinus. The symptoms of keratitis, or conjunctivitis, that appear suddenly in childhood, are often due to difficult dentition. Dr. Williams reported the following case in the *Dental Cosmos*: An alveolar asbcess from a superior molar discharged on the lower margin of the orbit, under the outer commissure of the eyelid. The pus had burrowed under the zygomatic process and temporal muscle and was prevented from pointing in the temple not only by those structures, but also by the strong temporal fascia. It had then passed through the spheno-maxillary fissure into the outer and lower part of the orbit, to discharge by means of a fistulous opening in the location described. Distinctly marked exophthalmus of the eye was present along with serous chemosis of the conjunctiva. After the pus had been evacuated by means of an incision in the temporal region, improvement immediately followed.

Some forms of nasal disease have been traced to diseased teeth, and, as regards antral disease, it is only necessary to mention the fact, admitted by all, that many serious cases have been cured by the removal of one or more diseased teeth or roots. Chorea, hysteria, epilepsy, paraplegia and insanity have been caused by dental irritation and disease. "The fact has long been established that there is great sympathy between the eyes, stomach, brain, teeth and the nervous system, and the pathological reactions, when the teeth and oral cavity are in a diseased conditions calls loudly to the understanding of these conditions by any who attempt to treat them." It has been said that every inspiration of a person with a diseased mouth carries septic matter to the lungs and in time might cause death. "The symptoms and treatment of the morbid conditions of the teeth themselves, and of the parts immediately in connection with them, are not satisfactorily managed by the M.D., and seldom does the D.D.S. properly treat remote nervous affections in various parts of the body, or constitutional derangements arising from these local causes; diagnosis is often difficult, but often possible between those affections which *are* and other very similar ones which can not be traced. Both dentistry and medicine are needed for the relief of physical suffering and the prevention of disease, and such results can be best reached in certain cases in proportion as dentist and physician are willing to consult and co-operate, for their work certainly overlaps." The minimum time required to graduate a physician is three years, and the same time is required of the dentist, so there must be considerable to dentistry after all. There can be no doubt if medical colleges would have their students study dental anatomy, pathology, etc., they would be able to diagnose and cure many cases in which they now fail. Sometimes an ulcerated tooth is diagnosed by physicians as a tumor, and is treated systemically, and, perhaps, locally until it points externally on the face or neck. "That living and dead tissue can be made to exist in the same organ in the system, without presenting to the patient or practitioner any special pathological condition, is an achievement in medical and surgical practice of which the dentists have a right to be proud."

Conservatism says that "a diseased part which can be restored to a healthy condition must not be sacrificed; thus, if the exposed or putrid pulp of a tooth is a disturbing element, the tooth need not be extracted." Small things, because of their size, are often unrecognized and their proper treatment overlooked, but they sometimes lead to the most confusing complications. A large tumor, readily detected by any one, requires far less learning to understand as a cause of suffering than does an apparently causeless pain which is finally shown to have been reflected by the sympathetic nervous system from a distant point of irritation.

Prof. G. L. Curtiss, the oral surgeon, says: "Among the causes of affections of the antrum, the teeth and their diseases stand first. The root of a tooth may merely penetrate, or it may extend into the antrum half of its length with only the mucous membrane intervening; if the pulp of such a tooth becomes putrid, the poisonous influence soon extends to the surrounding tissue. There is no resistance as when the root is enveloped in bone, consequently soreness of the tooth, one of the first symptoms of pericemental inflammation, may not be present. The nervous system is under a continuous strain where a purulent condition of the antrum exists.

"In two cases recently observed, one of the patients had shown marked symptoms of insanity, and the other was insane for three months of the twelve for several years prior to operation. Fistulous openings under the chin, on the neck, back and breast have been caused by teeth containing dead putrid pulps.

After two years' treatment for rheumatism of the left shoulder, the cause was found in an abscessed molar, which was treated and filled, when the pain subsided. By extracting the diseased root of a superior third molar, paralysis of that side of the face was cured.

"In a case of epilepsy which had been treated by different ones, it was found that the primary cause was four *badly* decayed teeth, two of them ulcerated and two with exposed pulps, for this reason there was a great drain upon the system and a continual nervous strain, and the food not being properly masticated indigestion brought on the attack of epilepsy. These cases show in what diverse directions reflexes from the teeth may ramify."

The medical profession has justly been jealous of their good repute, and are trying to live up to a higher standard of scientific attainment. If we are honest and possess good professional ability, the medical profession will be glad to clasp hands with us, as any calling which makes itself worthy of recognition is sure to be recognized. If a dentist wishes to succeed well, he must understand to a reasonable extent the workings of the whole human system, and the more he knows of its different functions the better he will be able to diagnose correctly the diseases with which he comes in contact; thus, from the supposition that he is learned in his professional specialty, it becomes his duty to himself and patients to study and develop his powers in the general branches of medicine; without these acquirements, he has not done "his highest" in developing by brain and hand-work those powers which give him the strength to withstand the test of time. Ought not the M.D. to have a fair comprehension of the teeth and their diseases? They are vitalized structures, supplied with arteries, veins and nerves, and through these nerves are connected in sympathy with all parts of the body. He should be able to relieve an aching tooth without resorting to extraction, but in saving the precious organ add to his fame by preserving the health, beauty and comfort of those who seek his counsel. Do not extract any tooth which causes pain, whether decayed or not, but remember where the permanent teeth are removed they do not appear again; nature does not, as in many parts of the body, supply the chasm which has been made. The temporary teeth are often extracted too soon, and they are sometimes mistaken for permanent ones by those who have had no training in dental anatomy; there is a duty and responsibility in these matters which ought not to be misunderstood. It is our belief that the most successful physicians are those who have made the teeth somewhat of a study, and when called to a case examine them carefully should there be the least cause to suspect their implication. At the September meeting of the Odontological Society of London, England, the point was raised as to the advisability of the medical council making it compulsory that every medical man should know something about the anatomy, physiology and pathology of the teeth. They also recommended consultation between physician and dentist, and suggested an interchange by reading papers before medical and dental societies. One gentleman present, an assistant surgeon to a general hospital, said he often found it desirable to consult with a dentist in regard to cases under his care. When a patient who is wearing the ordinary rubber plate complains to the physician of continual sore mouth, throat or indigestion, it would be well for him to have a consultation with the dentist. Take a case of neuralgia of the fifth pair of nerves, and generally the pain is located in the temple, cheek, mouth, jaws and teeth. Now point out the M.D. who diagnoses these cases properly and cures the greatest number, and we can tell to a certainty that he is one who does not fail to examine closely the mouth and teeth; he knows that pericementitis, arising from whatever cause, produces a very painful type of this disease; also that carious teeth are among the most prominent causes, and should receive his first attention, and he will almost invariably find them or their surrounding tissues in a diseased condition. The ideas we have given are practical and should tend to bring the M.D. and D.D.S. into a closer relationship of harmony. Let each be ready to assist the other in consultation or operation, and not regard any with jealousy or as rivals, but work together for the good of all humanity, for with such friendly feelings in your heart, success will in time surely crown your efforts.

DISCUSSION.

Dr. C. R. Butler, Cleveland: It is hardly necessary to add any more about the necessity or the advantage, in the practice of dentistry, of having a knowledge so that when we come in contact with other members of the general profession, we will be able to discuss physiologically and pathologically the question of therapeutics in an intelligent manner, especially in consultation, where it is deemed advisable and advantagous.

One of the difficulties that has presented itself to my mind has been the lack, perhaps, of self assertion on the part of those who engage in the practice of dentistry when they come in contact or association with those in the practice of general medicine, and they are disposed, perhaps, to take a back seat, or rather feel that they are not entitled to an opinion that is of just as much value as the men engaged in the general practice of medicine or any other specialty of the profession. There is no good reason why there should be any discrimination or recrimination between the two professions. We are all dependent upon one another, and the part that is engaged in the practice of medicine or any other specialty, if they get into trouble, they go to the dentist the same as we go to the general surgeon. If we are in trouble we seek the assistance of those who can help us out. If the physician has a pain in face or teeth he is not able to fathom, he will come to the dentist just as quick as the layman, and in this way there is the very best reason why there should be a degree of harmony and no disharmony with one who is carrying on practice in a different field from the one in which we are engaged. I think there is more of this disposition being shown all the way around—that is my experience and observation, and I think many here can voice the same thing.

Dr. H. A. Smith, Cincinnati: I am very glad to commend the paper for it is a very interesting one. The tenor of the paper indicates that there is disharmony between the medicine man and the tooth carpenter. I don't think it obtains very much between intelligent physicians and intelligent dentists. I think they are getting nearer together. Almost weekly we have patients referred to us by intelligent, bright physicians. It is no indication that they know nothing about dentistry themselves. On the other hand, we get cases we know nothing about and refer them to the physician. I have no degree of M.D., which qualifies a man to practice medicine, and I am not going to raise the question whether it is necessary to have the M.D. degree, but I would rather be a qualified man as a D.D.S. than to be a cross between them ; one of those double breasted fellows. Does the degree of M.D. stand for anything? There are many dentists who have the degree who don't deserve it very much. We should settle these questions among ourselves. M.D. means, from the medical standpoint, doctor of medicine. Long ago, in a discussion, Dr. ——, of Chicago, said the degree M.D. would be sufficient to stand for all. I advocate that we should have both degrees and have harmony between the dentists and the medical men.

I do not quite agree that there is this remarkable degree of want of harmony between the intelligent physician and the intelligent dentist.

Dr. Henry Barnes, Cleveland: I agree that there is not so much want of harmony as formerly between the two professions. I frequently meet medical gentlemen in consultation in relation to certain cases, neuralgia, and others of a similar kind.

The other day I saw a case and it is worth while to describe it, because we hear so much about painless dentistry. I had heard from time to time of a certain lady, who said she went to a distant city to have her dentistry done because it was painless. The case fell into the hands of a physician. She was troubled with severe neuralgia and he referred her to me to see if anything was wrong with her teeth. The mouth presented one of the worst cases I had ever seen. It certainly was not painless when I saw her. The trouble arose from an exposed nerve of the third superior molar which was extracted. I have not seen the patient since, but am sure the pain was lessened very materially, although there was enough other trouble in the mouth to have caused neuralgia. The teeth had evidently been scooped out saucer-shape and a great deal of cement put in. I will give you an illustration of how it had been done :

The second superior bicuspid was broken down on its mesial, distal and lingual surfaces and much of the root was lost. The cement was so placed as to impinge upon the disto-lingual angle of the first bicuspid and the mesia-lingual angle of the first molar, thus bridging over the interproximate space. This is a fair sample of the painless dentistry which had been done for her. Quite a few cases of antral disease have been treated for catarrh, which, when referred to the dentist, have been correctly diagnosed.

Dr. Grant Molyneaux, Cincinnati: I don't see that there is any lack of harmony between the dental and the medical profession. I don't see why we should be eternally running to doctors to tell us what is the matter when we can tell it ourselves. The paper had reference to a condition following the use of rubber dentures. If a dentist knows that the trouble is from the use of a rubber denture, why go to a doctor to consult about it. When he knows how to relieve it he doesn't need to go to the doctor. Suppose he does go to the physician for the sake of harmony, what can the doctor know about it? He can only diagnose it. He would say, there is a plate in the mouth, you had better see if it is not the plate that produces this condition. In the end he would go to the dentist to see if it was not the plate in the first instance, and if he found there was a local lesion, the dentist would refer the case to a doctor to take care of the lesion.

I think the paper was excellent, but I don't like to run after doctors.

About three weeks ago a lady met with a street car accident and had her face and lips cut. She called in an excellent surgeon and he didn't know what to do. He did not say he wanted a consultation with a dentist, but he said "you get a dentist." The lips were split and the teeth driven up into the nose and into the side of the mouth. The doctor had faith that the dental profession could take care of it. He didn't even sew up the lip, which was necessary, and the dentist had to stitch the lip, and she came to me, an every day dentist—not a double breasted one either—single breasted. A physician was not called in and she progressed rapidly, but, if he had attempted to operate there, the very certain result would have been the removal of every one of those teeth, and a great destruction, not only in the deformity of the mouth, but in the deformity of the face as well. There are many possibilities there. Possibility of necrosis following; possibility of sequestrum resulting from that, or, if there had been a consultation, in case of carelessness of the dentist treating the case. When called to my attention it took a couple of hours to find out the exact state of things. The teeth were pushed up clear out of sight. Most any surgeon would have amputated the whole thing, but this surgeon had faith in the D.D.S. profession. He did not say "go to a medical dentist," but he said, "get a dentist to fix that."

Dr. W. D. Snyder, Sidney: In my opinion, if any one feels that he is ignored or not treated right by his town physicians, it is largely his own fault. I have known a case or two of simple abscessed teeth being referred directly from the dentist to the physician. Perhaps to treat an abscessed tooth, he would fill a canal or fill up the tooth, and it would afterwards recur and bring a good deal of pain, and he would say, "you had better see your physician about that." Certainly the physician could not have very much faith in the dentist, and wouldn't very likely refer any one else back to him who could not see plain enough that the trouble had all come from that tooth.

There have been several little cases that have come under my observation. Cases of spasmodic closure of the jaws, coming from erupting wisdom teeth, and lesions of the mouth have been referred to me. Patients would go to a doctor before they consulted any dentist, and I have known them to be referred to some of my other dentists or friends and colleagues. I think the dentist that will try to educate himself in his line, will have no trouble with his physicians. I don't believe the dentist should try to cover the ground of dentistry and everything else. I think it is all right if he knows it, but to try to be both physician and dentist, I don't think it is hardly the right thing to do, but if anybody else gets along well with it it is no business of mine. So far as lesions of the mouth are concerned, he should be as well posted as possible, and try to take care of his business as well as he can.

Dr. H. A. Smith, Cincinnati: I was reminded during this discussion of a very remarkable book published lately by a dentist with only the degree of D.D.S. I mean Dr. Bodecker's work, "The Anatomy and Pathology of the Teeth." I think it is the greatest work of the last decade in dentistry. You know who Dr. Bodecker is? For the last ten years he has given his spare time to the making of this book, and would you say that he was not competent to take care of these cases? This shows that a thoroughly intelligent dentist is capable of producing a work that would be a credit to any department of the whole art. In the book is the celebrated case of Dr. Mahon, a millionaire dentist, the only millionaire dentist I think I know of. Young men, make a note of that. This is called the $10,000 case. The Doctor does not give the amount of money spent in it, for I suppose it would not be professional to mention the fee in a case like that. It is a case where a lady who had facial neuralgia involving the inferior nerve had spent a good deal of money with physicians and failing to get relief went to Paris and London for further treatment, and these treatments had cost $10,000. She applied to Dr. Mahon, (I don't know whether her common sense or some harmonious doctor sent her) he extracted the third molar and she got well immediately, and the Doctor added to his million by the operation, or ought to, at least in cases of that kind.

We could go on and tell case after case where doctors had made a mistake, but do you ever think dentists make mistakes? I think they do, I have, a good many of them. We must not charge everything to the doctor. The intelligent dentist, and especially the ignorant dentist, is always making mistakes.

I saw in the clinics the other day, in Cincinnati, a case of an abscessed tooth with an ulcer. This ulcer was the result of an abscess of the lower molar tooth. It was put into the hands of a woman studying dentistry and in a day or two she had a nice case of healing, and saved the tooth, showing what a woman of average intelligence in dentistry can do. This case had been treated for months by a prominent doctor.

Dr. F. E. Battershell, New Philadelphia: I can say that I was unexpectedly called to reply to a toast on a subject somewhat similar to this. I was attending a medical society in our town, and what little I had to say was about in this line: that when a physician finds a case of cleft palate it will be, perhaps, just as well for him to turn it right over to a dental surgeon and let him make an obturator for that case. It may save making an operation and lacerating those parts and having a failure on account of it. On the other hand, the dentist, after having filled the teeth and treated the pain to the best of his ability, perhaps finds that the patient needs other treatment, then I think it would be courteous to turn the patient over into the hands of the physician, and in that general way of exchange I think this harmony between physicians and dental surgeons should be kept up.

Dr. J. Taft, Cincinnati: It seems to me there ought to be no discord or disharmony between the dentist and physician any more than there would be disharmony between the oculist and aurist or any other specialist and the general practitioner. They are all engaged in the same line in the general way of practice, making remedies and preventing disease. There is no objection to any getting all the knowledge he can that will bear upon his practice. It is commendable to do whatever, can be made contributory to success in the treatment of these cases. He ought to get everything that is pertinent to, and could be subservient to the best ends in his practice, no difference whether practicing on the teeth or eye or skin or any other part of the human body.

What attainment is necessary to the practice of dentistry? Simply that a man shall have a knowledge of the foundation principles of medicine. The dental schools embrace the foundation principles of medical practice in their curriculum—anatomy, physiology, pathology. A state of inflammation in the gum is of the same nature as in the foot, stomach, uterus or anywhere else.

Some of our profession claim that each dentist ought to be a medical graduate—that is, possessed of medical attainments up to about the point of a medical practitioner. When a dentist does that, he makes his attainments equal to that of a medical man.

A great deal of stress is laid on M.D. and D.D.S., and such things. That is simply a certificate that A or B has done such and such work, and it is not worth making half so much of as many people make of it. It is the knowledge that ought to be attained. To know how and what to do in cases is the great point.

If you are very sick, in great danger, and call upon somebody to help you, do you call upon a man simply because he is a D.D.S. or an M.D.? No, because you know a many have this degree whom you would not trust in a dangerous case. A great many graduates from our medical colleges are no more fit for the general practice, though they have an M.D. degree, than an ordinary dentist—than many dentists that have no degree at all.

In this matter of consultation, what is the object? A has a case, a bad case, and wishes he knew more about it than he does. He confesses he don't know about the case and wishes to know what Dr. B says about it, and he asks for a consultation. He says: "I want to consult Dr. B and get his opinion about the case, and I want his suggestions about what ought to be done;" and he is called into consultation. The man is in the dark and wants some one to help him. Is there anything wrong in that? The patient would not object. I have gone to another dentist frequently to get help in a case in which I felt myself helpless. Many times I have been helped. If it is a case where one feels that a physician would help him, it is right and due to the patient that he secure such consultation. Wherever you have confidence you can receive aid, ask for it.

An intelligent dentist will never be repulsed by a physician in asking aid in the way of a consultation about a case. On the other hand, the physician often goes to the dentist for consultation. Dentists are more and more being called to occupy positions more closely allied to the physician than heretofore. Why? Because they are making advancement all the time—all the time knowing more and more by thorough study—by bringing to their aid everything that can be made contributory to their advancement. This ought and will be recognized. Dentists are occupying positions they formerly did not occupy. They frequently occupy positions in medical colleges and lecture in these colleges. Dentists are admitted into medical societies, even the highest medical societies in our country. In the local societies they are invited to membership and to read papers. It shows a closer and closer alliance and harmony and we ought to be gratified to see the day when we will not quibble and talk about M.D.'s, and D.D.S.'s.

DR. H. L. AMBLER, Cleveland: I have only a word to say and I don't wish to occupy any time in the discussion of this matter. I have heard the remarks of the gentlemen who preceded me and they agree with me exactly. I have no complaint to make of the treatment I have received from physicians or surgeons. I did not write the paper with the idea that there was a very large amount of disharmony, but there certainly is some, and it was with the idea of encouraging consultations and a working together of the two branches. One gentleman misunderstood the idea I wished to convey in speaking about rubber dentures. The remark was that where a patient had been wearing a rubber denture, and if a physician was called to treat some ailment that was induced by wearing rubber dentures, it would be his duty to consult a dentist.

